# Development and Comparative Evaluation of Two Different Label-Free and Sensitive Fluorescence Platforms for Analysis of Olaparib: A Recently FDA-Approved Drug for the Treatment of Ovarian and Breast Cancer

**DOI:** 10.3390/molecules28186524

**Published:** 2023-09-08

**Authors:** Ibrahim A. Darwish, Nasr Y. Khalil

**Affiliations:** Department of Pharmaceutical Chemistry, College of Pharmacy, King Saud University, P.O. Box 2457, Riyadh 11451, Saudi Arabia

**Keywords:** Olaparib, fluorescence-based techniques, microwell and HPLC analysis, green and high throughput approach

## Abstract

Olaparib (OLA) is a PARP inhibitor drug which has been recently approved by the Food and Drug Administration (FDA) for the treatment of ovarian and breast cancer. A convenient analytical tool for the quantitation of OLA in its dosage form and plasma samples was urgently needed. This study describes, for the first time, the development of two different label-free and sensitive fluorescence-based platforms for the pharmaceutical and bioanalysis of OLA. These platforms were microwell-assisted with a fluorescence microplate reader (MW-FLR) and high-performance liquid chromatography with fluorescence detection (HPLC-FD). Both MW-FLR and HPLC-FD employed the native fluorescence of OLA as an analytical signal. The MW-FLR involved measuring the fluorescence signals in 96-well white-opaque plates. The HPLC-FD involved chromatographic separation of OLA and duvelisib (DUV), as an internal standard on a Nucleosil-CN HPLC column (250 mm length × 4.6 mm i.d., 5 µm particle diameter) with a mobile phase composed of acetonitrile: water (25:75, *v/v*) pumped at a flow rate of 1.7 mL/min. Elution of OLA and DUV was detected using a fluorescence detector. The optimal conditions of both MW-FLR and HPLC-FD were established, and they were validated according to the guidelines of the International Council for Harmonization for the validation of analytical procedures. The linear ranges of MW-FLR and HPLC-FD were 25–1000 and 5–200 ng/mL, respectively, with limits of detection of 15 and 1.7 ng/mL, respectively. The accuracy and precision of both platforms were confirmed as the recovery values were ≥98.2% and the relative standard deviations (RSD) were ≤2.89%. Both methodologies were satisfactorily applied to the quantitation of OLA in its commercial dosage form (Lynparza^®^ tablets) and plasma samples with high accuracy and precision. The greenness of both MW-FLR and HPLC-FD was assessed using two different multiple parameter-based metric tools, and the results proved their greenness and adherence to the requirements of green analytical approaches. Both platforms have simple procedures and acceptable levels of analytical throughput. In conclusion, the proposed MW-FLR and HPLC-FD are valuable tools for routine use in quality control and clinical laboratories for the quantitation of OLA for the purposes of pharmaceutical quality control, pharmacokinetic studies, and bioequivalence testing.

## 1. Introduction

**Figure 1:**
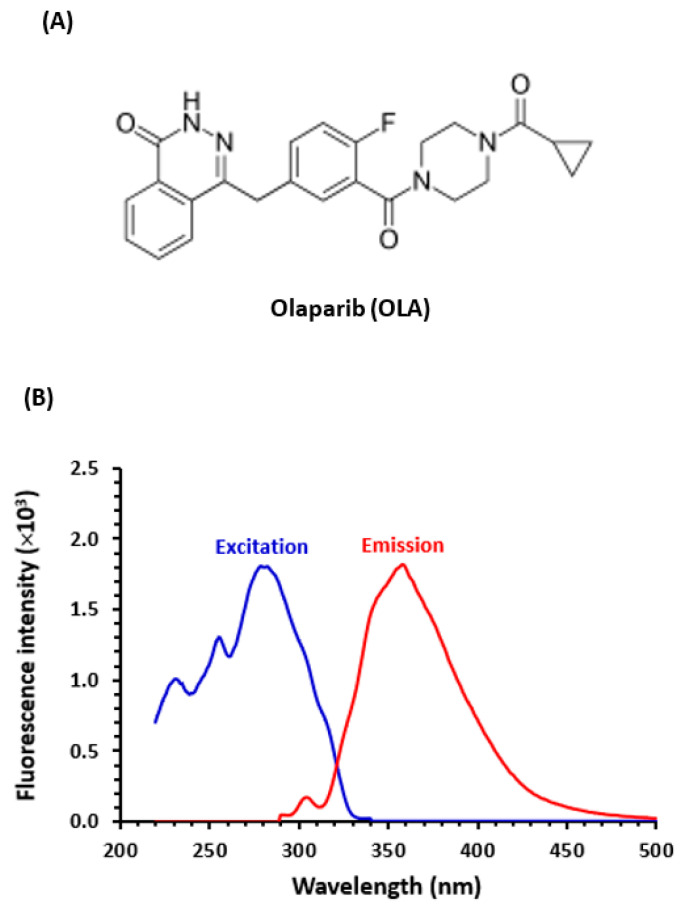
The chemical structure of Olaparib (**A**) and fluorescence spectra (excitation and emission) of its solution (**B**). The concentration of OLA used for recording the spectra was 10 µg/mL, in methanol).

Olaparib (OLA) is a small molecule that belongs to a novel class of anti-cancer drugs known as poly (ADP-ribose) polymerase (PARP) inhibitors. The chemical structure of OLA is given in Figure 1, and its chemical name is 4-[(3-[(4-cyclopropyl carbonyl) piperazin-1-yl]carbonyl)-4-fluorophenyl]methyl(2H)phthalazin-1-one. Its molecular formula is C_24_H_23_FN_4_O_3_ with a molecular weight of 434.5 g/mol [1,2]. OLA is effective in the treatment of certain types of cancer, particularly ovarian and breast cancer. It works by a selective potent blocking of the activity of PARP enzymes (PARP 1 and PARP2), which play a role in repairing damaged DNA in cells. The inhibition of PARP by OLA prevents cancer cells, particularly those with mutations in the BRCA genes, from repairing DNA damage. This leads to the accumulation of damaged DNA in cancer cells, resulting in their death. OLA is marketed as oral tablets under the trade name of Lynparza^®^ tablets (AstraZeneca Pharmaceuticals LP, Wilmington, DE, USA). It was approved by FDA on 11 March 2022 for the adjuvant treatment of adult patients with deleterious or suspected deleterious germline BRCA-mutated (gBRCAm) human epidermal growth factor receptor 2 (HER2)-negative high-risk early breast cancer who have previously received multiple lines of chemotherapy. It is also used for the maintenance treatment of adult patients with germline or somatic BRCA-mutated advanced epithelial ovarian, fallopian tube, gBRCAm metastatic pancreatic cancers who are in complete or partial response to first-line platinum-based chemotherapy, and those with homologous recombination repair (HRR) gene-mutated metastatic castration-resistant prostate cancer (mCRPC) who have progressed following prior treatment with enzalutamide or abiraterone [3,4]. In the case of ovarian cancer, OLA has shown remarkable efficacy, especially in patients with gBRCAg mutations. It has demonstrated the ability to significantly extend progression-free survival and delay the need for subsequent lines of chemotherapy. Furthermore, OLA showed impressive outcomes in the therapy of patients with advanced ovarian cancer who have responded to platinum-based chemotherapy. The recommended dose of OLA is 250 mg taken twice daily, which can be modified to manage adverse reactions, particularly in patients with moderate renal impairment [3].

While OLA has shown beneficial chemotherapeutic effects, it also showed some side effects including disorders of gastrointestinal, blood/lymphatic, nervous, metabolic, respiratory, and immune systems [3]. The incidence of these side effects and their frequency were related to drug exposure. Therefore, a thorough understanding of the link between drug content in the dosage form (Lynparza^®^ tablets) and patients’ blood is critical for its effective and safe administration. To achieve this goal, an efficient and reliable analytical tool is required for the quantitation of OLA in both tablets and plasma samples. The application of this tool in pharmaceutical quality control laboratories would ensure the exact OLA content in Lynparza^®^ tablets and its uniformity from tablet to tablet. This will ensure effective treatment with OLA. Also, the application of the tool in clinical laboratories will be useful for drug monitoring during therapy and, accordingly, enable the dose adjustment to meet specific conditions for particular patients.

The existing techniques for the detection or quantitation of OLA are limited; they include mass spectrometry imaging [5], HPLC coupled with an ultraviolet detector (HPLC-UV) [6,7], and liquid chromatography with tandem mass spectrometry (LC-MS/MS) [8,9,10,11,12,13]. Mass spectrometry imaging [5] was used for drug visualization in the different areas of tumor sections of mice tissues implanted with cancer cell lines. HPLC-UV [6,7] was used for the quantitation of OLA in its bulk drug and dosage forms; however, the sensitivity was inadequate for the analysis of plasma samples. LC-MS/MS [8,9,10,11,12] offered adequate sensitivity for the quantitation of OLA in plasma samples; however, the technique is inherently associated with some disadvantages including the high cost of acquiring and operating/maintaining the instrument, the complexity of instrument operation, the challenge of establishing optimal separation conditions and ionization techniques, and the limited throughput for processing many samples in a reasonable time period. To overcome these drawbacks, the development of a better alternative methodology was essential.

Fluorescence and fluorescence spectrometry hold great significance in various scientific disciplines and practical applications. They allow for the precise and sensitive analysis of fluorescent molecules or compounds, providing valuable information about their properties, interactions, and concentrations. In the field of chemistry, fluorescence spectrometry aids in the characterization of compounds, the identification of unknown substances, and the monitoring of chemical reactions. In biochemistry and molecular biology, it plays a vital role in studying biomolecules, such as proteins and nucleic acids, their structure, dynamics, and interactions. In medical research and diagnostics, fluorescence spectrometry enables the detection of disease markers, the analysis of cellular processes, and the monitoring of drug responses. Its non-invasive nature, high sensitivity, and versatility make fluorescence spectrometry an indispensable tool in research, industry, and healthcare, contributing to advancements in diverse fields and improving our understanding of the molecular world.

Fluorescence-coupled analytical techniques, particularly microwell-based analysis assisted by a fluorescence microplate reader (MW-FLR) and HPLC with fluorescence detection (HPLC-FD) have great importance in the field of pharmaceutical and biomedical analysis because they provide extraordinary advantages over LC-MS/MS [14,15,16]. MW-FLR offers high sensitivity, simplicity of procedures, capability for automation, and high throughput [15]. HPLC-FD also offers high sensitivity, a lower cost of acquiring and operating the instrument, and simpler optimization of chromatographic conditions and detection parameters [16].

This study describes, for the first time, the development of two different fluorescence-based platforms for the quantitation of OLA in both dosage form (Lynparza^®^ tablets) and plasma samples. These platforms are MW-FLR and HPLC-FD, and they are characterized by high sensitivity, simple procedures, greenness, and high throughput.

## 2. Results and Discussion

### 2.1. Strategy of Study and Fluorescence Spectra

The therapeutic benefits of OLA and the urgent need for an accurate and simple tool for its quantitation in Lynparza^®^ tablets and plasma samples were the factors behind its selection for this study. The fluorometric techniques involving measuring the native fluorescence of drug molecules have been widely used and offer several advantages, thus they were considered in the present study.

The chemical structure of OLA (Figure 1A) contains an aromatic 2(H)-phthalazine-1-one moiety, which is a rigid moiety, connected to 4-cabonyly-4-fluorophenyl, which is also an aromatic and conjugated structure. From these structural features, it was expected that OLA emits a strong native fluorescence. This assumption was confirmed by its fluorescence spectra generated in the laboratory (Figure 1B). The spectra showed that OLA has maximum excitation and emission wavelengths at 280 and 360 nm, respectively. The intense native fluorescence of OLA was encouraging for its employment in the development of sensitive fluorescence-based platforms for its direct quantitation in both tablets and plasma samples.

### 2.2. Development of MW-FLR

The following section describes the optimization of different factors affecting the relative fluorescence intensity (RFI) of OLA solution and ultimately the analysis sensitivity.

#### 2.2.1. Selection of Microwell Plates

Black and white, transparent- or opaque-bottomed 96-well plates were available for fluorescence measurements; however, white plates have some advantages over the black ones. White plates generally provide higher signal intensity compared to black plates, resulting in increased excitation of fluorophore and enhanced signal intensity. White plates also offer better signal-to-background ratios than black plates do. The white background of wells minimizes the background fluorescence or autofluorescence, which can interfere with the detection of low-level fluorescence signals, resulting in the enhancement of the sensitivity and accuracy of fluorescence measurements. Furthermore, white plates offer better visualization of sample volume because their white background provides a high-contrast visual reference, making it easier to observe and monitor any changes in the sample, such as color shifts or precipitate formation [17]. For these advantages, white plates were selected for the development of the present MW-FLR for OLA. The fluorescence microplate reader, which is available in the laboratory, features a top-detection mode, thus white plates with opaque bottoms were selected in the present work.

#### 2.2.2. Effect of Solvent

**Figure 2:**
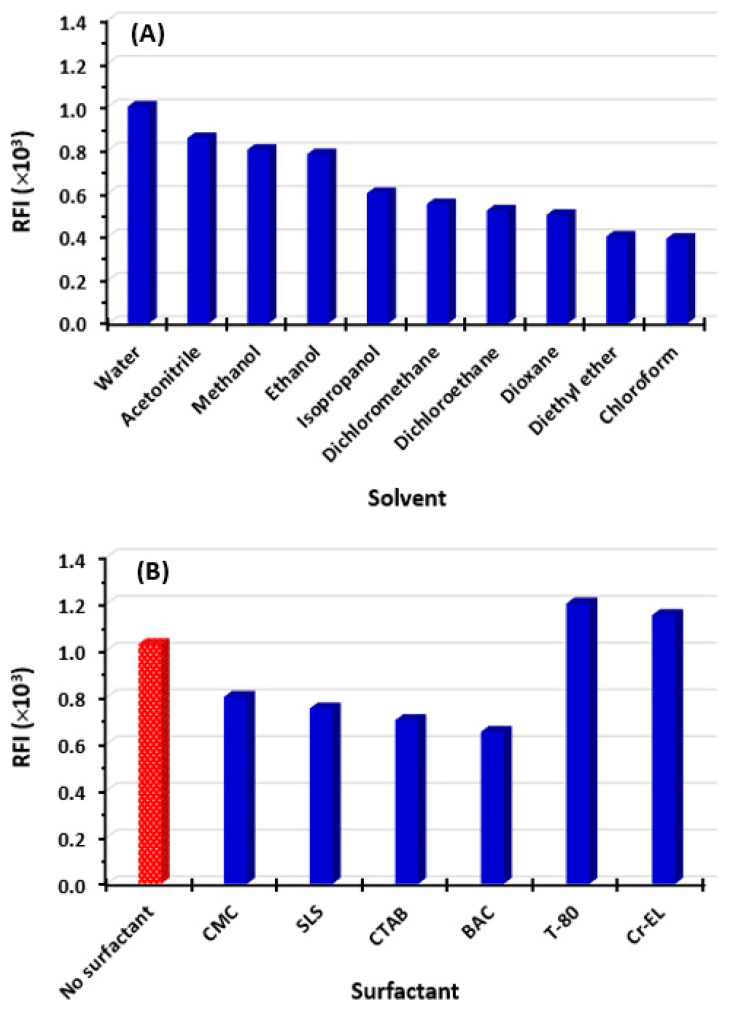
The effect of solvent (**A**) and type of surfactant (**B**) on RFI of OLA solution (5 µg/mL in methanol). Surfactants were carboxymethylcellulose (CMC), sodium lauryl sulfate (SLS), cetyltrimethylammonium bromide (CTAB), benzalkonium bromide (BAC), Tween-80 (T-80), and cremophor EL (Cr-EL).

The impact of solvent type on the RFI of OLA solution was investigated by measuring RFI in different solvents. These solvents were water, acetonitrile, methanol, ethanol, isopropanol, dichloromethane, dichloroethane, dioxane, diethyl ether, and chloroform. The results revealed that high RFI values were attained in solvents with a high polarity index, such as water, acetonitrile, methanol, and ethanol (Figure 2A). Lower RFI values were attained in solvents with a lower polarity index, such as isopropanol, dichloromethane, and dichloromethane (Figure 2B). This variation in RFI with solvent polarity was attributed to the varying strengths of interactions between OLA and solvents. Additionally, the OLA molecule possesses asymmetrical dipoles and exhibits different relaxation rates in various solvents. The arrangement of fluorophore in the excited state and the potential formation of hydrogen bonding can also impose effects on the fluorescence of OLA [18]. Among the superior solvents, water was chosen for all subsequent experiments due to its cost-effectiveness and, importantly, its environmental and health safety, aligning with sustainable and green analytical approaches.

#### 2.2.3. Effect of Surfactant

Previous studies have noted that incorporating a surfactant into a drug solution can form micelles and amplify its fluorescence intensity, thereby improving the sensitivity [19]. Therefore, various surfactants were investigated for their impact on the RFI of OLA. These surfactants included anionic, cationic, and non-ionic surfactants. The anionic surfactants were sodium lauryl sulfate (SLS) and carboxymethyl cellulose (CMC). The cationic surfactants were cetyltrimethylammonium bromide (CTAB) and benzalkonium bromide (BAC). The non-ionic surfactants were Tween-80 (T-80) and chromophor EL (Cr-EL). The results indicated that non-ionic surfactants gave a higher RFI than the ionic ones and water solution without any surfactant (Figure 2B). The fluorescence enhancement effect of non-ionic surfactants was attributed to excellent solubilization capabilities for hydrophobic compounds like OLA. By solubilizing the OLA molecules efficiently, non-ionic surfactants ensure a higher concentration of OLA molecules in the solution, thereby increasing its fluorescence intensity [20]. In comparison with water without any surfactant, the fluorescence enhancement by non-ionic surfactants was not significantly high, at ~20% (Figure 2B). In order to simplify the procedures, the inclusion of a surfactant was disregarded for all subsequent experiments.

#### 2.2.4. Effect of pH

**Figure 3:**
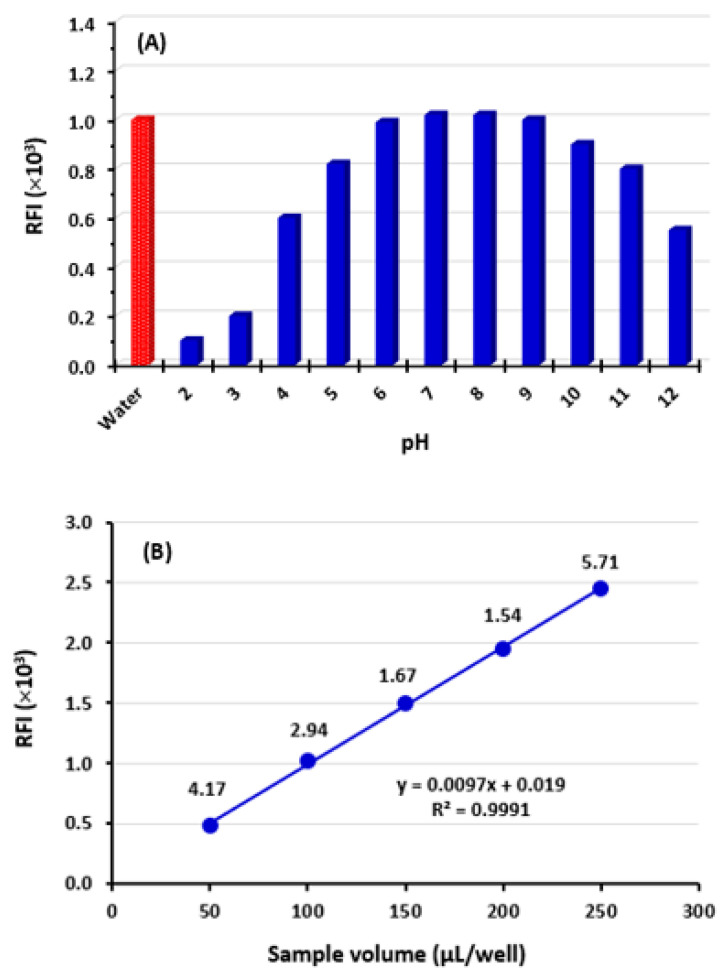
The effect of pH of buffer solution (**A**) and sample volume (**B**) on RFI of OLA solution (5 µg/mL in methanol).

The influence of pH on the RFI of OLA solution was studied by adding Britton–Robinson buffer solution of different pH values (pH 2–12) to its solution and subsequently measuring the RFI values. The findings (Figure 3A) revealed that highly acidic solutions (pH 2–4) and highly alkaline solutions (pH 12) had a quenching effect on the fluorescence of OLA solution. These effects were attributed to different possible mechanisms. Firstly, at high acidic pH values, the nitrogen atoms in the fluorophoric moiety of the OLA molecule (Figure 1A) could undergo protonation, leading to a decrease in fluorescence. The fluorescence quenching effect caused by the electron delocalization on the fluorophore, which decreased its susceptibility to excitation by the incident light [21]. Secondly, at high alkaline pH, OLA could potentially degrade, resulting in reduced fluorescence. Additionally, the pH variations could also impact intermolecular and intramolecular interactions, as documented in previous studies [21]. The highest RFI values were achieved in the pH range of 6–9, and these values were comparable with those achieved in non-buffered solution (Figure 3A). Consequently, all subsequent experiments were conducted without the addition of a buffer solution to the OLA solution. This approach offered several advantages, including a simple procedure, reduced costs, environmental friendliness, and safety considerations.

#### 2.2.5. Effect of Sample Solution Volume

To determine the optimal volume of OLA samples for analysis, different volumes (50, 100, 150, 200, and 250 µL/well) were dispensed into each well of the analysis plate. RFI values of these volumes were measured, and their relative standard deviations (RSD) were calculated. The findings indicated a direct correlation between the volume of OLA solution and RFI, as depicted in Figure 3A. Also, the precision of the measurements, as indicated by their RSD values, was improved (decreased RSD) as the sample volume increased up to 200 µL/well; however, low precision was observed with 250 µL/well. The best precise results (lowest RSD value of 1.54%) were obtained when a volume of 200 µL/well was utilized. Consequently, all subsequent experiments were conducted using a volume of 200 µL/well.

A summary of the optimization of variables influencing the fluorescence intensity of OLA and the optimum values selected for development of the proposed MW-FLR method are summarized in Table 1.

### 2.3. Development of HPLC-FD

#### Optimization of Chromatographic Conditions

As mentioned above, OLA exhibited intense native fluorescence (Figure 1B), thus it was explored for detection in the present study. The fluorescence spectra encompass maximum excitation and emission at 280 and 360 nm, respectively. Therefore, detection was set at these wavelengths. The optimization of chromatographic conditions aimed to identify the most suitable mobile phase, column, and flow rate. Initially, the separation was initiated using an isocratic elution mode on a C18 column (150 mm length × 3.9 mm i.d., 5 μm particle diameter) connected to a guard column. The column temperature was maintained at a constant 25 ± 2 °C. Separation experiments were conducted using a mobile phase composed of acetonitrile:water (50:50, *v*/*v*), with a flow rate of 1.0 mL/min. However, these preliminary conditions resulted in a quiet long run time (~20 min), which is not convenient for pharmaceutical and clinical applications and the quantitation of OLA in dosage form and plasma samples. This long run time (slow elution) was attributed to the high lipophilicity of OLA. To overcome this problem, a more polar cyano (CN) column was tested instead, and the mobile phase composition was adjusted to acetonitrile:methanol:water (40:20:40, *v*/*v*). These modifications significantly reduced the run time (retention time was ~3 min), which is very close to the expected retention time of endogenous plasma-containing fluorescent molecules. To achieve better elution, different mobile phase compositions and flow rates were evaluated. Optimal resolution, along with good peak shape, were obtained when the mobile phase consisted of acetonitrile:water (25:75, *v*/*v*) and the flow rate was set to 1.7 mL/min.

**Figure 4:**
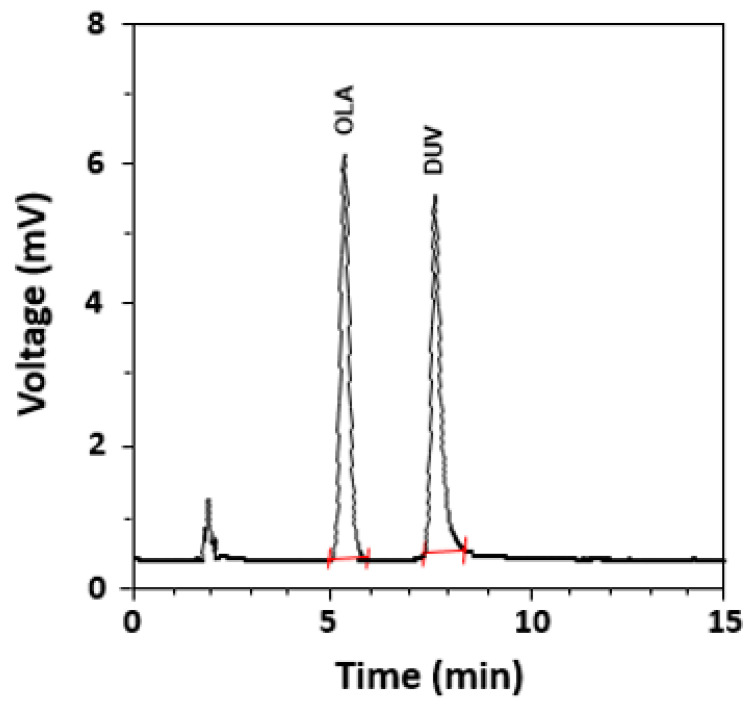
The chromatogram standard solutions of OLA and DUV (IS). The concentrations of OLA and DUV were 50 and 150 ng/mL, respectively.

In a subsequent set of experiments, other reversed phase columns, including C18 and phenyl columns with varying dimensions manufactured by different manufacturers, were tested. It was found that the Nucleosil-CN column (250 mm length × 4.6 mm i.d., 5 µm particle diameter) manufactured by Macherey-Nagel GmbH & Co. (Düren, Germany) provided the best chromatographic conditions for the elution of OLA. The run time was ~10 min and the retention time of OLA was ~5 min. In our ongoing research involving anti-cancer drugs, it was observed that some drugs were natively fluorescent molecules and emit their fluorescence at, or very close to, the emission wavelength of OLA. These drugs were seleciclib, linafinib, and duvelisib (DUV), thus they were tested to select one of them as an internal standard (IS). The results revealed that DUV was the best one because it gave an appropriate resolution from OLA, a good peak shape, and short run time without the need for changing the chromatographic conditions. The conditions were further refined for the separation of OLA and DUV (IS); the refined optimum conditions are summarized in Table 1. Under these optimized conditions, an acceptable resolution between OLA and DUV was achieved and gave sharp, narrow, and symmetric peaks at 5.2 and 7.6 min for OLA and DUV, respectively (Figure 4).

The system suitability and effectiveness were assessed, and various chromatographic parameters such as capacity factor, separation factor, resolution factor, peak symmetry factor, and number of effective theoretical plates were measured. The results of these evaluations are presented in Table 2.

### 2.4. Validation of MW-FLR and HPLC-FD

#### 2.4.1. Linear Range and Sensitivity

**Figure 5:**
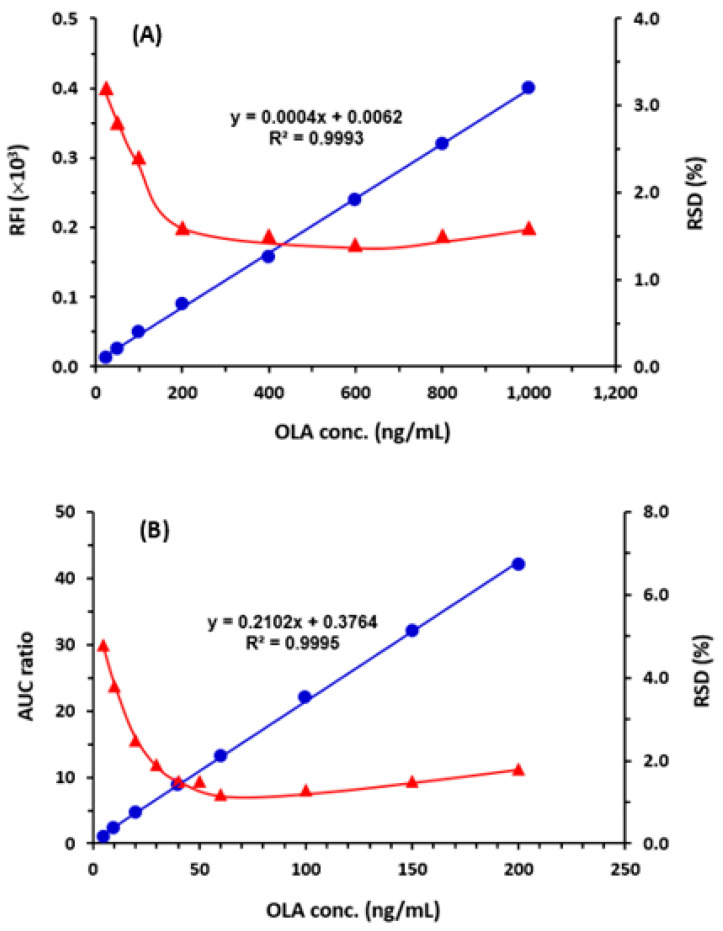
The calibration curves (●) and precision profiles (▲) for quantitation of OLA by the proposed MW-FLR (**A**) and HPLC-FD (**B**).

Calibration curves were generated under the refined optimal conditions for both MW-FLR and HPLC-FD (Figure 5), and the data were analyzed by least-squares of linear regression. The relationships between signals and their corresponding OLA concentrations were linear in the ranges of 25–1000 and 5–200 ng/mL, for MW-FLR and HPLC-FD, respectively. The determination coefficients (r^2^) of the linear equations were 0.9993 and 0.9995 for MW-FLR and HPLC-FD, respectively. The other calibration parameters for both methods are given in Table 3.

The limits of detection (LOD) and limits of quantitation (LOQ) were determined according to the International Council for Harmonization (ICH) guidelines [22]. The LOD values attained were 15 and 1.7 ng/mL, and LOQ values were 45 and 5 ng/mL for MW-FLR and HPLC-FD, respectively (Table 3).

#### 2.4.2. Precision and Accuracy

The precision profiles of both MW-FLR and HPLC-FD over their entire linear ranges are presented in Figure 5 as RSD (%). As shown, the RSD values were ≤3.4 and 4.5%, respectively. In subsequent separate sets of experiments, both intra- and inter-day precisions and accuracy were assessed at three concentration levels (low, medium, and high); these concentrations are shown in Table 4. RSD and recovery (%) with error (%) were used as measures for evaluating the precision and accuracy, respectively. For MW-FLR, the RSD values were 0.82–1.84 and 1.28–1.86% for intra- and inter-day precisions, respectively. For HPLC-FD, the RSD values were 1.42–1.82 and 1.84–2.21% for the intra- and inter-assay precisions, respectively. These small RSD values proved the high precisions of both MW-FLR and HPLC-FD.

The accuracy of both MW-FLR and HPLC-FD was evaluated using recovery studies at the same levels of concentration that were used for precision evaluation. The recovery values were 98.2–101.5% and 97.6–102.4% for MW-FLR and HPLC-FD, respectively, indicating the accuracy of both methods. Also, the error (%) was in the ranges of −1.81–1.52 and −1.52–2.42% for MW-FLR and HPLC-FD, respectively. These results confirmed an acceptable and comparable accuracy of both MW-FLR and HPLC-FD.

#### 2.4.3. Robustness and Intermediate Reproducibility

The robustness of an analytical method refers to the ability of the method results to remain unaffected by small variations in the internal method parameters [22]. MW-FLR involved the direct measurements of the native fluorescence of OLA solution without any extra experimental manipulations or conditions (e.g, pH, temperature, etc.), therefore the method is considered intrinsically robust. The robustness of HPLC-FD was assessed by conducting the analysis under slightly varying conditions (±10% of the predefined optimal condition) and determining the recovery value in each condition. The involved conditions were mobile phase composition ratio and flow rate. It was found that the recovery values ranged from 98.6–101.2 (±1.21–1.54%), confirming the robustness of HPLC-FD method and its convenience for routine application for the analysis of OLA.

The intermediate reproducibility of a method refers to the stability of the method results to remain unaffected when external factors such as analyst, laboratory, instrument, and days are varied [22]. The ruggedness of both MW-FLR and HPLC-FD was evaluated in terms of analyst-to-analyst and day-to-day variation. It was found that the RSD values did not exceed 2.82 and 3.13% for MW-FLR and HPLC-FD, respectively. These low RSD values revealed the intermediate reproducibility of the results of both methods.

### 2.5. Applications to the Analysis of Tablets and Plasma Samples

The aforementioned validation results assumed the applicability of both MW-FLR and HPLC-FD for the analysis of OLA in its tablets and plasma samples. This expectation was supported by the high sensitivity achieved by both platforms (low LOQ: 45 and 5 ng/mL for MW-FLR and HPLC-FD, respectively). These LOQ values are significantly lower than the reported mean maximum plasma concentrations (C_max_) of OLA, which are 5.4 and 7.6 μg/mL following a 300 mg dose once and twice daily, respectively [3]. Both MW-FLR and HPLC-FD were applied to the quantitation of OLA in commercialized Lynparza^®^ tablets and OLA-spiked plasma samples.

In the case of analysis by MW-FLR, the achieved mean recovery values of the nominated OLA concentrations were 100.7 ± 1.07 and 101.0 ± 1.16% for the analysis of tablets and plasma, respectively (Table 5).

**Figure 6:**
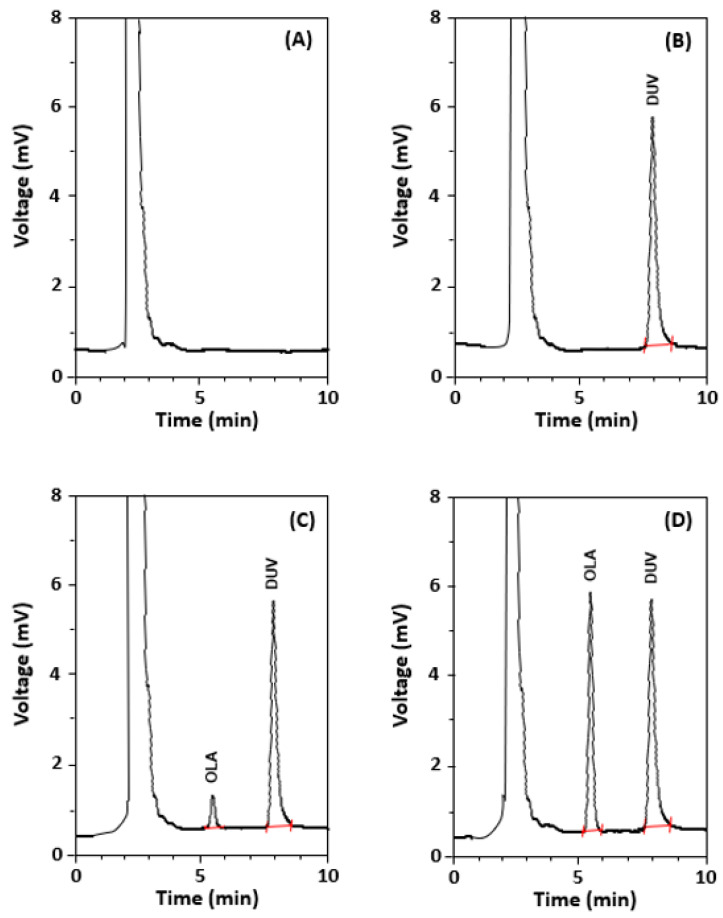
Representative chromatograms from (**A**) OLA-free blank human plasma, (**B**) plasma spiked with DUV (150 ng/mL), (**C**) plasma spiked with LOQ value of OLA (5 ng/mL) and DUV (150 ng/mL), and (**D**) plasma spiked with OLA (50 ng/mL) and DUV (150 ng/mL). mV is the detector response in millivolts.

Before analysis by HPLC-FD, the selectivity of the method was assessed by analysis of blank OLA-free plasma samples, and the experiments were conducted as guided by the ICH guidelines for the validation of analytical procedures [22]. Figure 6 showed representative chromatograms generated in four different conditions (Figure 6, panels A, B, C, and D). These conditions were (A): OLA-free sample (no DUV, no IS), (B): a plasma sample spiked with IS (DUV) at a concentration of (150 ng/mL), (C): a plasma sample spiked with both OLA at its LOQ concentration (5 ng/mL) and IS at a concentration of (150 ng/mL), and (D): a sample spiked with both OLA at a concentration (50 ng/mL) and IS at a concentration of (150 ng/mL). As shown in all chromatograms (Figure 6, panels A–D), no apparent interfering peaks were found at the corresponding retention times of either OLA or IS. In addition, no carryover was observed for both OLA and IS in plasma samples, as evident from the absence of any interference peaks following the injection of plasma samples containing OLA at its LOQ level. In a subsequent set of experiments, plasma samples spiked with three varying nominated concentration levels of OLA (40, 80, and 160 ng/mL) were prepared and analyzed using HPLC-FD. The achieved mean recovery values of the nominated OLA concentrations were 99.3 ± 1.94% (Table 5). This result revealed that both MW-FLR and HPLC-FD methods are appropriate for the routine accurate determination of OLA in QC plasma samples.

### 2.6. Greenness Levels of MW-FLR and HPLC-FD

**Figure 7:**
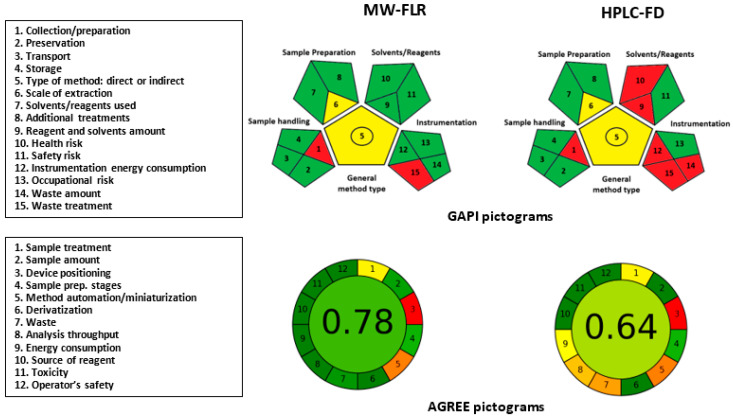
Evaluation parameters (in boxes on left hand side) and pictograms of GAPI and AGREE metric tools for evaluation of the greenness of the proposed MW-FLR and HPLC-FD for quantitation of OLA.

Green analytical chemistry (GAC) is a relatively new emerging approach that seeks to minimize the impact of analytical chemistry on the environment and human health. This approach emphasizes the use of analytical techniques that are more sustainable and environmentally friendly, including the reduction of hazardous chemicals, wastes, and energy consumption [23]. In the pharmaceutical field, GAC is becoming increasingly important as researchers and companies seek to develop more sustainable analytical processes in manufacturing and quality control. GAC practices in the pharmaceutical industry provide several advantages such as minimizing solvent use, reducing waste, and using green analytical techniques [24,25,26,27]. To accurately evaluate the greenness levels of MW-FLR and HPLC-FD and judge how they meet the requirements of GAC principles, two different multiple parameters-based metric tools were utilized, which provide precise and comprehensive assessments of the greenness of analytical procedures. These metric tools were the Green Analytical Procedure Index (GAPI) [28] and Analytical Greenness (AGREE) [29]. The details of the assessment steps of these tools have been described in their corresponding articles. The results obtained from both GAPI and AGREE tools regarding all the evaluation parameters are presented as pictograms (Figure 7).

In GAPI pictograms of both MW-FLR and HPLC-FD, parameters 1 and 15 (corresponding to sample collection/preparation and waste treatment, respectively) were given a red color in the pictograms. This red color assignment was given because samples collection/preparation was carried out in an off-line manner and the waste was not treated. Parameter 5 (corresponding to type of method) was assigned yellow in both MW-FLR and HPLC-FD because both are of the same type (direct quantitative methods). Similarly, parameter 6 (corresponding to scale of sample extraction) was assigned yellow in both methods because the samples extraction was performed identically in a microscale manner in both methods. The other evaluation parameters were given a green color in MW-FLR because they meet the tool greenness requirements. For HPLC-FD, these were given green or red colors depending on whether they meet the greenness requirements or not, respectively. Conclusively, both MW-FLR and HPLC-FD are green; however, the greenness level of MW-FLR is higher than that of HPLC-FD.

In AGREE pictograms, parameter 1 (corresponding to sampling procedure) was assigned with a yellow color in both MW-FLR and HPLC-FD because the samples treatment was manually carried out for both methods. Similarly, parameter 3 (corresponding to device positioning either on-line or off-line) was given a red color because the analysis was conducted using both a microplate reader and an HPLC system in an off-line way. Parameter 5 (corresponding to method automation/miniaturization) was orange in both MW-FLR and HPLC-FD because the analysis with both methods was conducted in a semi-automated way. The other parameters were assigned green for MW-FLR, and varying colors (green to orange) for HPLC-FD according to the tool guidelines [29]. The total scores of MW-FLR and HPLC-FD were 0.78 and 0.64, respectively. These scores confirmed an acceptable greenness level of both methods, although MW-FLR had a higher greenness level than HPLC-FD.

In overall conclusion, the results of GAPI and AGREE tools confirmed the greenness, with different levels, of both MW-FLR and HPLC-FD, and their adherence to GAC principles.

### 2.7. Comparison of MW-FLR with HPLC-FD

The analytical performances of both MW-FLR with HPLC-FD were compared in terms of the following parameters:

*Sensitivity*: HPLC-FD offered higher sensitivity compared to MW-FLR; their LODs were 1.7 and 15 ng/mL, respectively (Table 3). The higher sensitivity of HPLC-FD was attributed to the separation and concentration of OLA, which can enhance the LOD. On the other hand, MW-FLR may have limitations in sensitivity due to factors such as background fluorescence from the microplate and the need for higher sample volumes. Additionally, the powerful design of fluorescence detector in HPLC-FD might also contribute to its higher sensitivity than the chromatic detection system of the fluorescence microplate reader.

*Linear range*: MW-FLR covered a wider linear range than HPLC-FD; the ranges were 25–1000 and 5–200 ng/mL, respectively (Table 3). The wider range of MW-FLR was attributed to the accommodation of a larger sample volume (200 µL/well), rather than the sample injection volume limitations encountered in HPLC-FD. The wide range of MW-FLR is beneficial for samples with a large concentration difference between samples, particularity in pharmacokinetic and bioequivalent studies of OLA.

*Accuracy and precision*: Both MW-FLR and HPLC-FD offered acceptable accuracy and precision; however, MW-FLR had better precision (lower RSD) than HPLC-FD. RSD values were 0.82–1.86 and 1.42–2.21% for MW-FLR and HPLC-FD, respectively (Table 4). The better precision of MW-FLR was attributed to the use of microplates and parallel processing of samples, reducing variability due to inter-assay variations. Moreover, replicates within the microwell plate can improve the intra-assay precision. On the other hand, the relatively low precision of HPLC-FD might be due to slight fluctuations in the chromatographic conditions such as column temperature and/or flow rate during the chromatographic runs.

*Throughput*: MW-FLR offered higher throughput compared to HPLC-FD due to the ability to analyze multiple samples simultaneously in a 96-well microplate format. Additionally, the throughput of MW-FLR can be enhanced to increase the number of samples processed without significantly increasing the experimental time of the two approaches. The first one is the use of high-density microplate formats, such as 384- or 1536-well plates instead of traditional 96-well plates. The second approach is the automation of the analysis by using automated liquid handling systems or robotic platforms to perform sample dispensing, reagent addition, and plate washing. Automation reduces manual handling and speeds up the process, allowing for higher throughput. On the other hand, HPLC-FD, having a short run time, has a throughput property but is at a lower level than MW-FLR. It was possible to enhance the throughput of HPLC-FD by increasing the flow rate or using a shorter column; however, care should be taken to avoid any possible deterioration of the resolution efficiency.

*Greenness*: The comprehensive evaluation of the greenness of MW-FLR and HPLC-FD by two multiple parameters-based metric tools revealed that MW-FLR had a higher greenness level than HPLC-FD. The higher greenness level of MW-FLR was attributed to two main reasons, which are the used volume of solvents and energy consumption by the instruments. First, MW-FLR used smaller volumes of samples and solvents compared to HPLC, leading to potentially reduced waste generation; however, HPLC-FD required larger volumes of solvents for mobile phase, and column cleaning which had a negative impact on its greenness level. Second, the energy consumption of the fluorescence reader (in MW-FLR) was significantly lower than that of the HPLC system (in HPLC-FD), which consumes more energy due to the operation of pumps, the column temperature control oven, and the detector.

In summary, both MW-FLR and HPLC-FD offered comparable accuracy. MW-FLR offered a wider linear range, better precision, higher throughput, and higher greenness level than HPLC-FD. On the other hand, HPLC-FD offered higher sensitivity than MW-FLR. These data demonstrate that the format of the analytical method may influence its performance characteristics (sensitivity, precision, etc.), even when exactly the same signal is employed.

### 2.8. Advantages of MW-FLR and HPLC-FD over LC-MS/MS

In general, fluorescence-coupled analytical techniques have great importance in the field of pharmaceutical and biomedical analysis because of their extraordinary sensitivity, simplicity, and low cost as compared to LC-MS/MS. The proposed MW-FLR offers several advantages over the existing LC-MS/MS for OLA. These advantages include: (1) higher throughput, as MW-FLR was designed for a high-throughput analysis that could simultaneously analyze multiple samples of OLA in a microplate format, allowing for rapid analysis of large sample sets; (2) a comparable high sensitivity and selectivity of MW-FLR to those of LC-MS/MS; and (3) simpler and more straightforward MW-FLR procedures compared to those of LC-MS/MS. HPLC-FD also offered several advantages over LC-MS/MS. These advantages are: (1) being less expensive because the instrument and its operational costs are lower; (2) being faster than most reported LC-MS/MS methods because it involves simple sample preparation procedures and short chromatographic run time; and (3) the optimization of chromatographic conditions and fluorescence detection is simpler than those involved in LC-MS/MS.

## 3. Experimental

### 3.1. Apparatus

A fluorimeter, model: FP-8200 (JASCO Co., Ltd., Kyoto, Japan), was used for the recording of fluorescence spectra. Spectra Manager^®^ software was used to convert all recorded spectra to ASCII format. A microwell fluorescence reader, model FLx800 (Bio-Tek Instruments Inc., Winooski, VT, USA), was used and was operated by KC junior software, provided with the instrument. An HPLC system equipped with an auto-sampler and a fluorescence detector (Shimadzu Corporation, Kyoto, Japan) was operated, and data were acquisitioned using CLASS-VP software, version 5.032 (Shimadzu Corporation, Kyoto, Japan). A Purelab Flex water purification system (ELGA Veolia Ltd., High Wycombe, UK) was also used.

### 3.2. Chemicals and Materials

Olaparib (OLA) and duvelisib (DUV) were purchased from LC Laboratories (Woburn, MA, USA); their purity was >99%. Lynparza^®^ tablets (AstraZeneca Pharmaceuticals LP, Wilmington, DE, USA) were kindly donated by the Saudi Food and Drug Authority (SFDA: Riyadh, Saudi Arabia), labelled to contain 150 mg of OLA per tablet. Corning^®^ 96-well solid white flat bottom polystyrene microplates were purchased from Thermo Fisher Scientific (Waltham, MA, USA). A Nucleosil-CN HPLC column (250 mm length × 4.6 mm i.d., 5 µm particle diameter) and a guard column were obtained from Macherey-Nagel GmbH & Co. (Düren, Germany). Britton–Robinson buffer was composed of 0.04 M boric acid, 0.04 M phosphoric acid, and 0.04 M acetic acid adjusted at pH range (2–12) by using 0.2 M sodium hydroxide. Buffer components, surfactants, and Finnpipette^®^ adjustable single and 8-channel pipettes were obtained from Sigma-Aldrich Corporation (St. Louis, MO, USA). Blank drug-free human plasma was obtained from the Blood Bank of King Khalid Hospital (King Saud University, Riyadh, Saudi Arabia) and was kept frozen at −20 °C until used in the analysis. All solvents were of spectroscopic grade (Merck, Darmstadt, Germany). All other chemicals used throughout the work were of analytical grade. Distilled-deionized water was obtained from the Purelab flex water purification system.

### 3.3. Preparation of Standard and Sample Solutions

#### 3.3.1. Standard Solutions

An accurate quantity (10 mg) of standard material of OLA was transferred to a 5 mL volumetric flask. The material was dissolved in 1 mL of dimethyl sulfoxide (DMSO) and the volume was completed with methanol. This stock solution (2 mg/mL) was further diluted with water and HPLC mobile to give working solutions in the range of 25–1000 and 5–200 ng/mL for analysis by MW-FLR and HPLC-FD, respectively. These standard solutions were used for the generation of the calibration curves from which the unknown OLA concentrations in its samples (tablets or plasma) were calculated.

#### 3.3.2. Tablets Sample Solution

Ten Lynparza^®^ tablets were crushed in a mortar and transformed into a fine powder. Capsules’ contents were taken, combined, and weighed. An accurate quantity of the powder to 50 mg OLA was transferred to a 25 mL volumetric flask. Two mL of DMSO was added and the contents were well shaken and subsequently 20 mL methanol was added. The contents of the flask were subjected to sonication for 10 min to ensure complete dissolution of OLA. The volume was completed to the mark with methanol and filtered through 0.2 µm Millipore filter paper. This solution (2 mg/mL) was diluted with water and HPLC mobile to give working solutions in the range of 25–1000 and 5–200 ng/mL for analysis by MW-FLR and HPLC-FD, respectively. These sample solutions were analyzed by both MW-FLR and HPLC-FD, and their OLA concentrations were derived from the linear calibration equations, generated by using standard calibrator solutions of standard solutions of OLA.

#### 3.3.3. Plasma Samples

For analysis by MW-FLR: Aliquots (1 mL) of the plasma samples were mixed with equal volumes of methanol, vortexed for 30 seconds, and centrifuged for 10 min at 13,000 rpm by Biofuge Pico centrifuge (Heraeus Instruments, Germany). The supernatants were withdrawn using syringes fitted with 0.2 µm Millipore filters. The supernatants were diluted with water to give concentrations in the range of 25–1000 ng/mL. These solutions were analyzed by MW-FLR, and their OLA concentrations were derived from the calibration curves generated by using standard solutions of OLA.

For analysis by HPLC-FD, plasma samples were spiked with DUV (internal standard) and treated as described above regarding the protein precipitation and subsequent filtration. The supernatant, obtained by filtration, was diluted with HPLC mobile phase to give OLA concentrations in the range of 5–200 ng/mL and a fixed concentration of DUV (50 ng/mL). These sample solutions were analyzed by HPLC-FD, and their OLA concentrations were derived from the linear calibration equations, generated by using standard calibrator solutions of standard solutions of OLA.

### 3.4. Analysis by MW-FLR and HPLC-FD

For MW-FLR: a standard or sample solution of OLA (200 µL) was transferred to 96-microwell plates. The fluorescence of solutions was measured at 360 nm for emission and 280 nm for excitation.

For HPLC-FD: chromatographic analysis was conducted on a Shimadzu HPLC system equipped with an auto-sampler and a fluorescence detector. The HPLC column was Nucleosil-CN (250 mm length × 4.6 mm i.d., 5 µm particle diameter), kept constant at 25 ± 2 °C. Chromatographic resolutions were conducted in an isocratic mode with a mobile phase composed of acetonitrile:water (25:75, *v/v*) pumped at a flow rate of 1.7 mL/min. Aliquots (10 μL) of the samples (standards, tablets, or plasma) were injected into the HPLC system by the autosampler. Elution of the compounds was monitored by the fluorescence detector (set at 280 and 360 nm for excitation and emission, respectively). The relation between the peak area ratio of OLA to DUV peaks and the OLA concentration was used as the basis for the quantification.

## 4. Conclusions

For the first time, two different fluorescence-based analytical platforms were developed for the validation of the pharmaceutical and bioanalysis of OLA. These platforms were MW-FLR and HPLC-FD. The analytical performances of both methods were compared, and the data revealed that both MW-FLR and HPLC-FD had comparable accuracy. MW-FLR was superior to HPLC-FD in terms of having a wider linear range, better precision, higher throughput, and higher greenness level; however, HPLC-FD was superior to MW-FLR in terms of the higher sensitivity. These data demonstrate that the format of analytical method may influence its performance characteristics (sensitivity, precision, etc.), even when the same analytical signal is employed. The proposed MW-FLR and HPLC-FD are valuable for routine use in quality control and clinical laboratories for the quantitation of OLA for the purposes of quality, pharmacokinetic studies, and bioequivalence testing. The choice between MW-FLR and HPLC-FD depends on the availability of the instrument and the number of samples being measured. In summary, the proposed platforms can be applied in broader fields of cancer research, enabling high-throughput analysis, biomarker discovery, and the study of tumor microenvironment dynamics. Their application in these broader fields of cancer research enhances our understanding of the disease, facilitates drug development, and potentially leads to improved diagnostic and therapeutic approaches.

## Figures and Tables

**Table 1:** Optimization of experimental conditions for the MW-FLR and HPLC-FD for determination of OLA.

Method/Condition	Studied Range	Optimum Value
MW-FLR		
Solvent	Different ^a^	Water
Surfactants (1%, *w*/*v*)	Different ^b^	None
Buffer pH	2–12	None
Volume of sample (µL/well)	50–250	200
Excitation wavelength (λ_ex_, nm)	220–340	280
Emission wavelength (λ_em_, nm)	390–500	360
HPLC-FD		
Organic solvent in a mobile phase	Acetonitrile, methanol	Acetonitrile
Acetonitrile:water ratio (%)	50:50–20:80	25:75
Column	Different ^c^	Cyano
Flaw rate (mL/min)	0.5–2	1.7
Internal standard	Different ^d^	Duvelisib
Excitation wavelength (λ_ex_, nm)	220–340	280
Emission wavelength (λ_em_, nm)	390–500	360

^a^ Solvents tested were water, acetonitrile, methanol, ethanol, isopropanol, dichloromethane, dichloroethane, dioxane, diethyl ether, and chloroform. ^b^ Surfactants used were carboxymethylcellulose (CMC), sodium lauryl sulfate (SLS), cetyltrimethylammonium bromide (CTAB), benzalkonium bromide (BAC), Tween-80 (T-80), and cremophor EL (Cr-EL). ^c^ Column tested were C18-, phenyl-, and Cyano-based columns with different dimensions. ^d^ Internal standards tested were seleciclib, linafinib, and duvelisib.

**Table 2:** Chromatographic parameters for OLA and DUV (IS) by the proposed HPLC-FD.

Parameter	Value
Retention time of OLA (min)	5.2 ± 0.2
Retention time for DUV (min)	7.6 ± 0.3
Capacity factor (*K*′)	5.84
Separation factor (α)	1.25
Resolution factor (Rs)	2.24
Peak asymmetry factor	1.51
Number of theoretical plates (N) per meter	1162

**Table 3:** Regression and statistical parameters for the determination of ALC by the proposed MW-SFL and HPLC-FD.

Parameter	Value	
	MW-FLR	HPLC-FD
Linear range (ng/mL)	25–1000	5–200
Intercept	6.2	0.3764
Slope	4	0.2102
Determination coefficient (r^2^)	0.9993	0.9995
Limit of detection (ng/mL)	15	1.7
Limit of quantitation (ng/mL)	45	5.0

**Table 4:** Precision and accuracy of MW-FLR and HPLC-FD for the quantitation of OLA.

OLA Concentration (ng/mL)	Intra-Day ^a^		Inter-Day ^b^	
	Recovery (% ± RSD)	Error (%)	Recovery (% ± RSD)	Error (%)
MW-SFL				
50	100.5 ± 0.82	0.52	99.8 ± 1.45	−0.22
500	98.2 ± 1.84	−1.81	101.5 ± 1.86	1.52
800	99.4 ± 1.17	−0.62	98.6 ± 1.28	−1.41
HPLC-FD				
10	102.2 ± 1.82	2.21	102.4 ± 2.21	2.42
100	98.5 ± 1.64	−1.52	99.6 ± 2.13	−0.41
180	101.4 ± 1.42	1.41	102.9 ± 1.84	2.89

^a^ Average of 3 determinations. ^b^ Average of 6 determinations.

**Table 5:** Applications of MW-FLR and HPLC-FD for the quantitation of OLA in Lynparza^®^ tablets and plasma samples.

Nominated OLA Concentration (ng/mL)		Recovery (% ± RSD) ^a^	
		MW-FLR	HPLC-FD
Lynparza^®^ tablets			
200		99.8 ± 2.38	99.6 ± 1.18
400		100.5 ± 2.20	98.9 ± 3.05
800		101.9 ± 1.26	100.7 ± 3.85
	Mean	100.7 ± 1.07	99.7 ± 0.91
Plasma samples			
40		99.8 ± 3.47	101.4 ± 2.84
80		102.1 ± 2.02	98.8 ± 3.17
160		101.2 ± 2.53	97.6 ± 4.38
	Mean	101.0 ± 1.16	99.3 ± 1.94

^a^ Average of 3 determinations.

## Data Availability

All data are in the article.

## Sample Availability

Samples of the compounds are available from the authors.
